# Supplementary material for: Seeds Priming with Melatonin Improves Root Hydraulic Conductivity of Wheat Varieties under Drought, Salinity, and Combined Stress
Source: Int J Mol Sci. 2024 May 6;25(9):5055. doi: 10.3390/ijms25095055 (PMC11084420; doi:10.3390/ijms25095055)
Supplement: Supplementary file 1 [file ijms-25-05055-s001.zip › ijms-2969832-supplementary.pdf]

Supplementary Results

1. The effects of seed-priming on root water potential and relative conductivity in shoot

As shown in Figure S4, compared to no-priming treatment, water-priming and melatonin-priming treatments significantly increased root water potential of H4399 under CK, PEG, NaCl, and PEG + NaCl conditions. In Y1212, by comparison, water-priming and melatonin-priming treatments greatly increased root water potential only under NaCl and PEG + NaCl stresses. However, compared to no-priming treatment, water-priming and melatonin-priming treatments insignificantly affected on root water potential of X19 under PEG and NaCl stresses. While two priming treatments strongly increased root water potential of X19 under PEG + NaCl stress.

From Figure S5**A**, relative to no-priming treatment, water-priming treatment significantly reduced the relative conductivity in shoot of H4399 under PEG, NaCl, and PEG + NaCl stresses, by 8.91%, 3.95%, and 3.26%, respectively. Under CK and PEG conditions, there was no significant difference between no-priming treatment and melatonin-priming treatment in relative conductivity of H4399. By comparison, melatonin-priming treatment markedly decreased relative conductivity in shoot of H4399 under NaCl and PEG + NaCl stresses, by 3.95% and 5.56%, respectively. In Y1212, compared to no-priming treatment, water-priming treatment obviously reduced the relative conductivity in shoot under PEG, NaCl, and PEG + NaCl stresses, by 2.42%, 4.10%, and 1.74%, respectively (Figure S5 **B**). And melatonin-priming treatment greatly reduced it only under NaCl stress by contrast (Figure S5 **B**). In X19, water-priming and melatonin-priming treatments significantly reduced the relative conductivity in shoot under CK, PEG, NaCl, and PEG + NaCl conditions (Figure S5 **C**).

The effects of seed-priming on expression of key genes regulating melatonin synthesis in roots

As shown in Figure S7, compared to no-priming treatment, in H4399 and Y1212, water-priming and melatonin-priming treatments significantly increased expression levels of *TaSNAT* and *TaASMT2* under CK, PEG, NaCl, and PEG + NaCl conditions. And by comparison, water-priming treatment elevated more expression levels of these genes in H4399 and Y1212 than that in melatonin-priming treatment. However, by comparison, in H4399, water-priming and melatonin-priming treatments significantly reduced expression levels of *TaASMT1* under CK, PEG, and NaCl conditions. In X19, by contrast with no-priming treatment, the transcript levels of *TaCOMT* was greatly reduced by water-priming treatment under PEG stress, yet increased by water-priming treatment under PEG + NaCl stress. And melatonin-priming treatment extremely elevated the expression levels of *TaCOMT* under CK condition in compared with no-priming treatment.

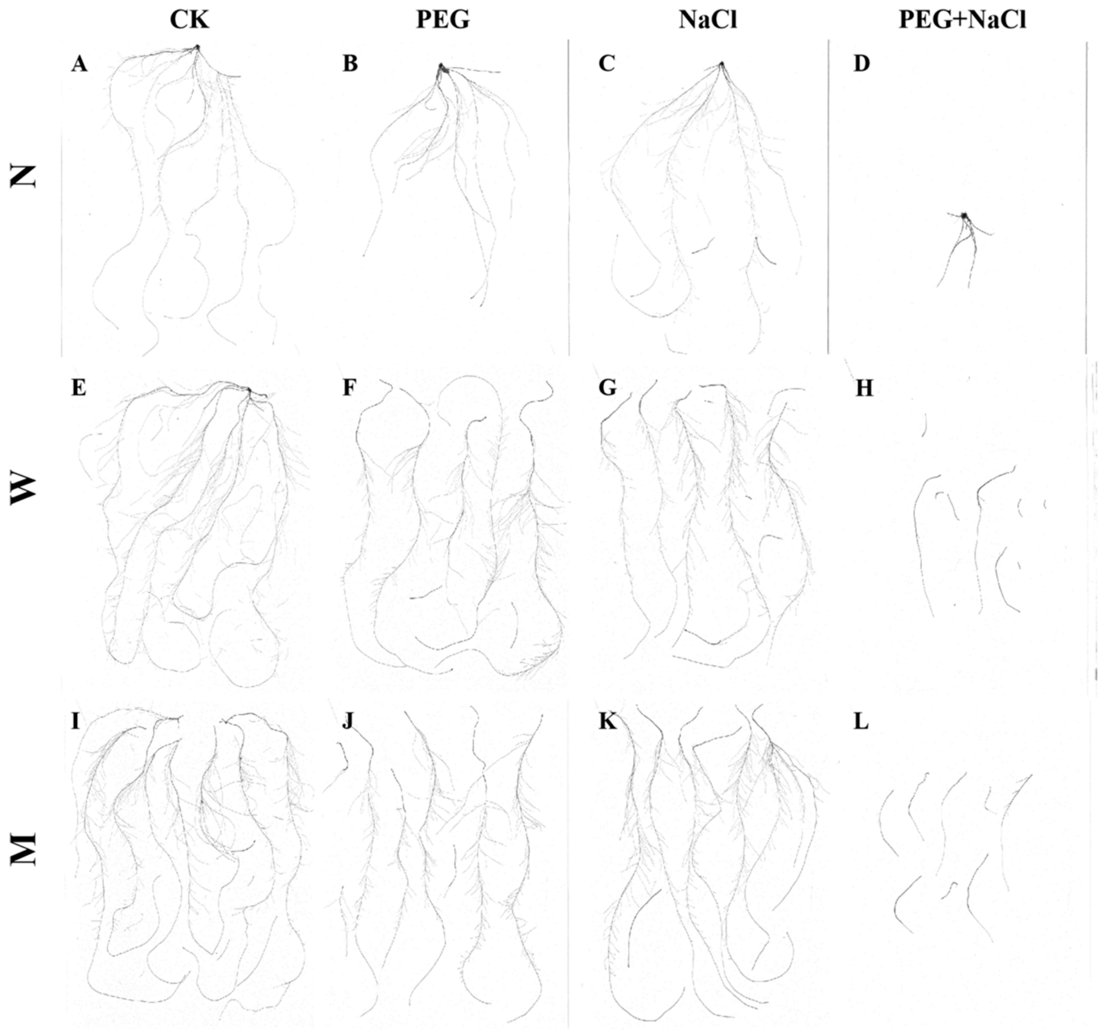

Figure S1. The root morphology of H4399 from no priming (N), water priming (W), and melatonin priming (M) under PEG, NaCl, and PEG + NaCl treatment. **A**, **E**, and **I** represent the CK; **B**, **F**, and **G** represent the PEG treatment; **C**, **G**, and **K** represent the NaCl treatment; **D**, **H**, and **L** represent the PEG + NaCl treatment; **A**, **B**, **C**, and **D** represent the no priming (N) treatment; **E**, **F**, **G**, and **H** represent the water priming (W) treatment; **I**, **J**, **K**, and **L** represent the melatonin priming (M) treatment.

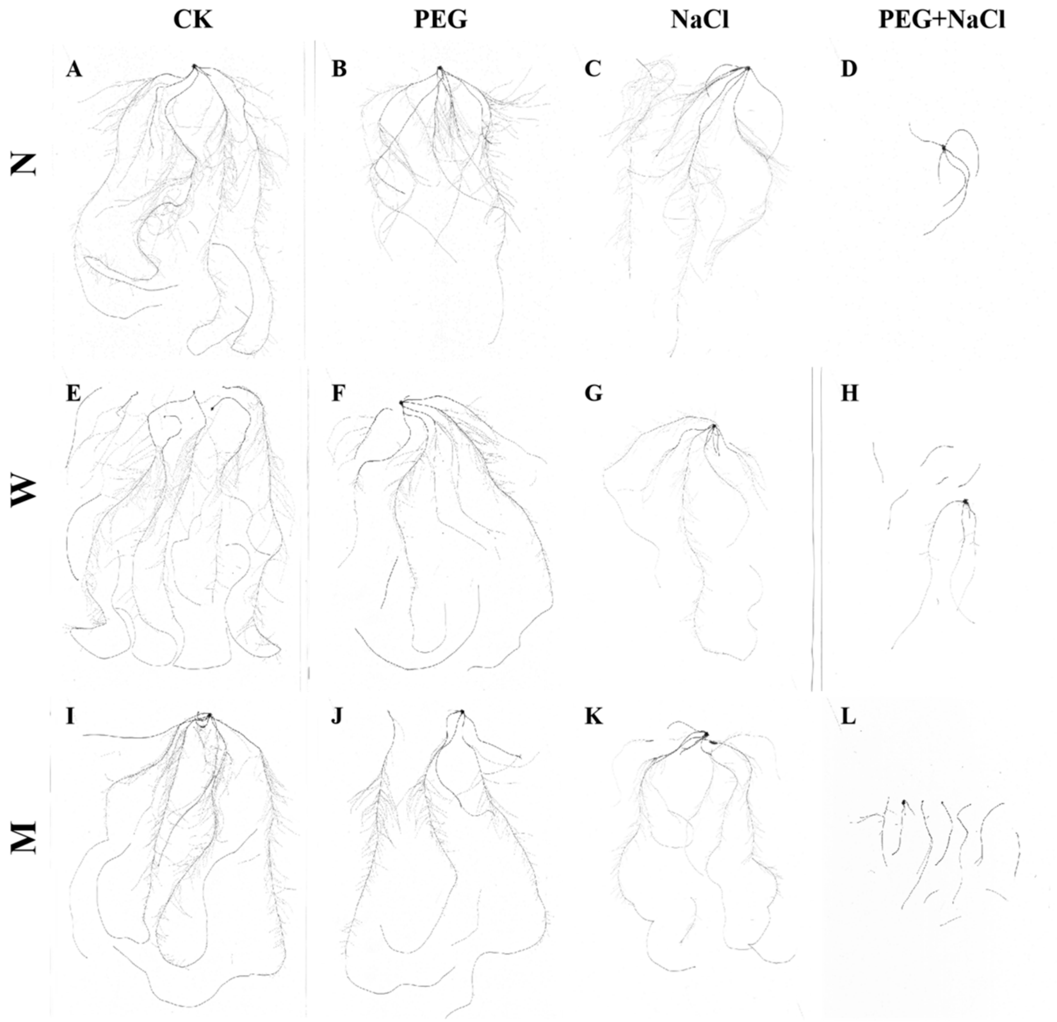

Figure S2. The root morphology of Y1212 from no priming (N), water priming (W), and melatonin priming (M) under PEG, NaCl, and PEG + NaCl treatment. The root morphology of H4399 from no priming (N), water priming (W), and melatonin priming (M) under PEG, NaCl, and PEG + NaCl treatment. **A**, **E**, and **I** represent the CK; **B**, **F**, and **G** represent the PEG treatment; **C**, **G**, and **K** represent the NaCl treatment; **D**, **H**, and **L** represent the PEG + NaCl treatment; **A**, **B**, **C**, and **D** represent the no priming (N) treatment; **E**, **F**, **G**, and **H** represent the water priming (W) treatment; **I**, **J**, **K**, and **L** represent the melatonin priming (M) treatment.

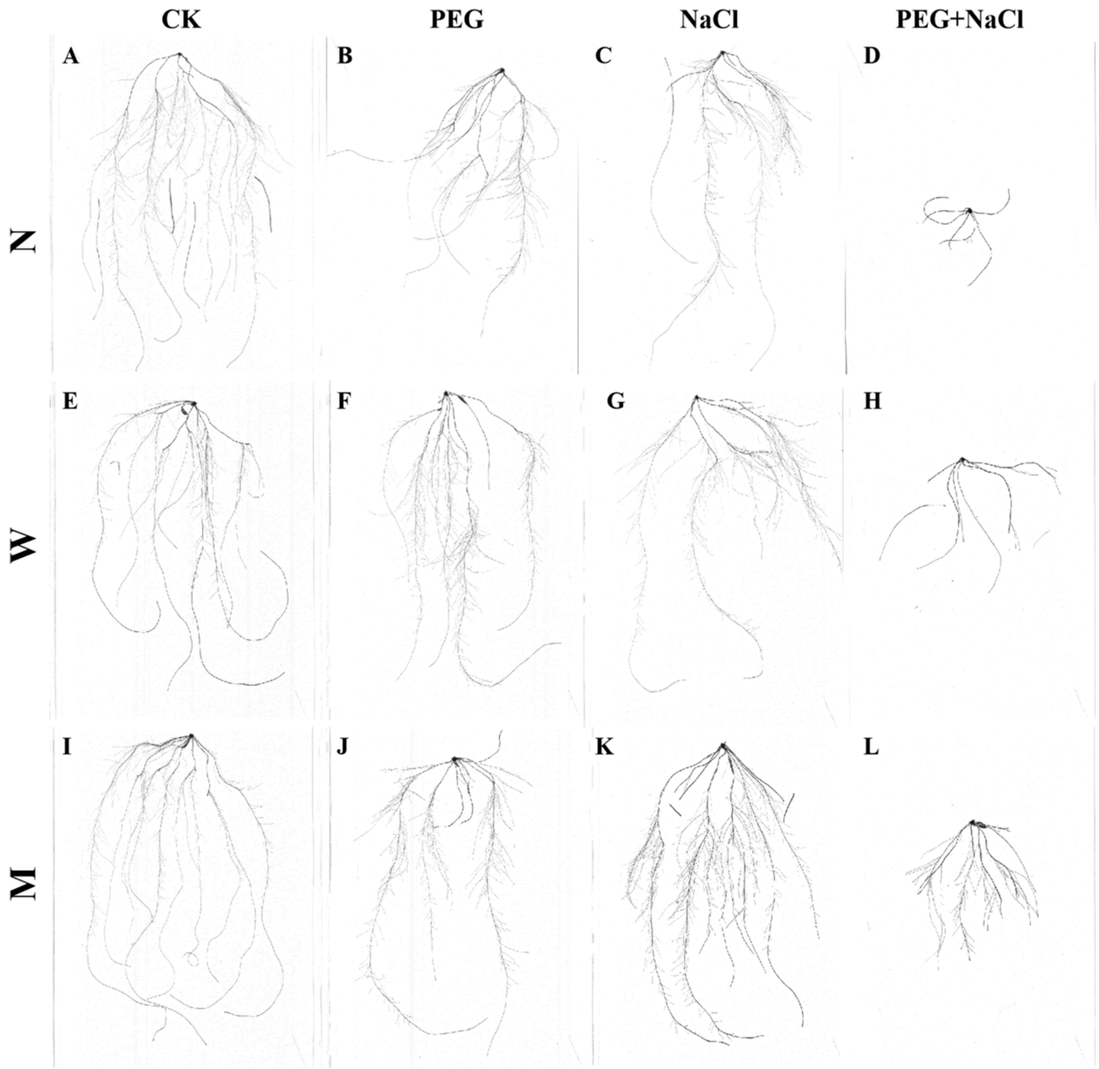

Figure S3. The root morphology of X19 from no priming (N), water priming (W), and melatonin priming (M) under PEG, NaCl, and PEG + NaCl treatment. **A**, **E**, and **I** represent the CK; **B**, **F**, and **G** represent the PEG treatment; **C**, **G**, and **K** represent the NaCl treatment; **D**, **H**, and **L** represent the PEG + NaCl treatment; **A**, **B**, **C**, and **D** represent the no priming (N) treatment; **E**, **F**, **G**, and **H** represent the water priming (W) treatment; **I**, **J**, **K**, and **L** represent the melatonin priming (M) treatment.

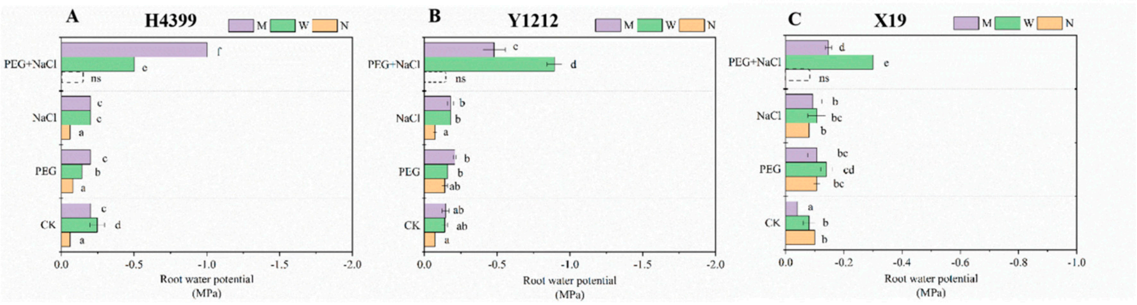

Figure S4. The root water potential of three wheat seedlings from no priming (N), water priming (W), and melatonin priming (M) under PEG, NaCl, and PEG + NaCl treatment. The standard deviation (SD) value from three replicates is represented as error bars in figures and different lowercase letters indicate significant differences at the 0.05 level.

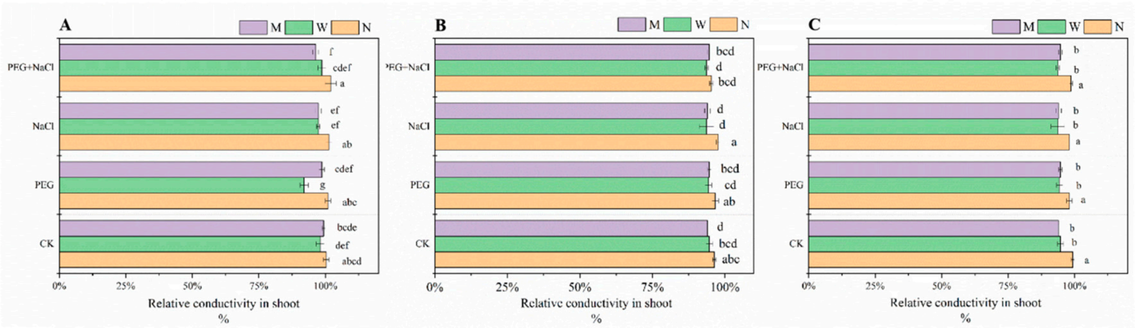

Figure S5. The relative conductivity in shoot of three wheat seedlings (**A** H4399, **B** Y1212, and **C** X19) from no priming (N), water priming (W), and melatonin priming (M) under PEG, NaCl, and PEG + NaCl treatment. The standard deviation (SD) value from three replicates is represented as error bars in figures and different lowercase letters indicate significant differences at the 0.05 level.

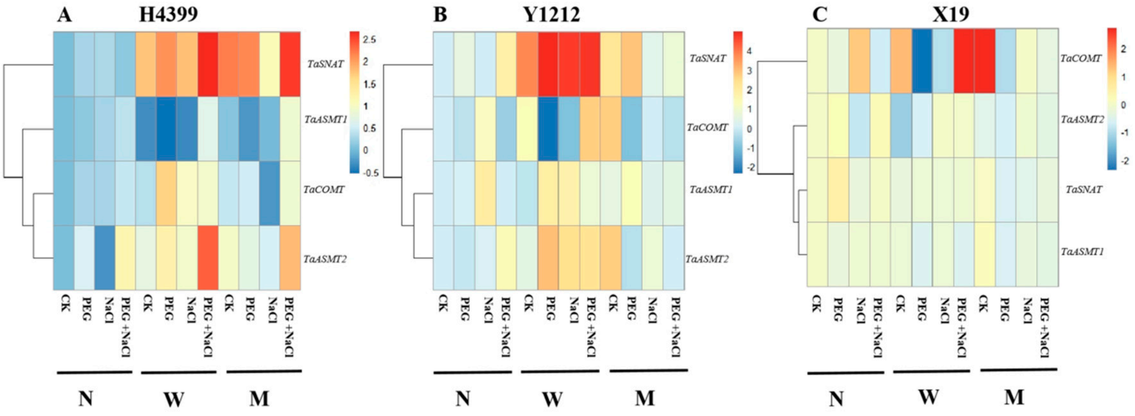

Figure S6. The heat map analysis of some genes regulating melatonin synthesis in root of three wheat seedlings from no priming (N), water priming (W), and melatonin priming (M) under PEG, NaCl, and PEG + NaCl treatment.

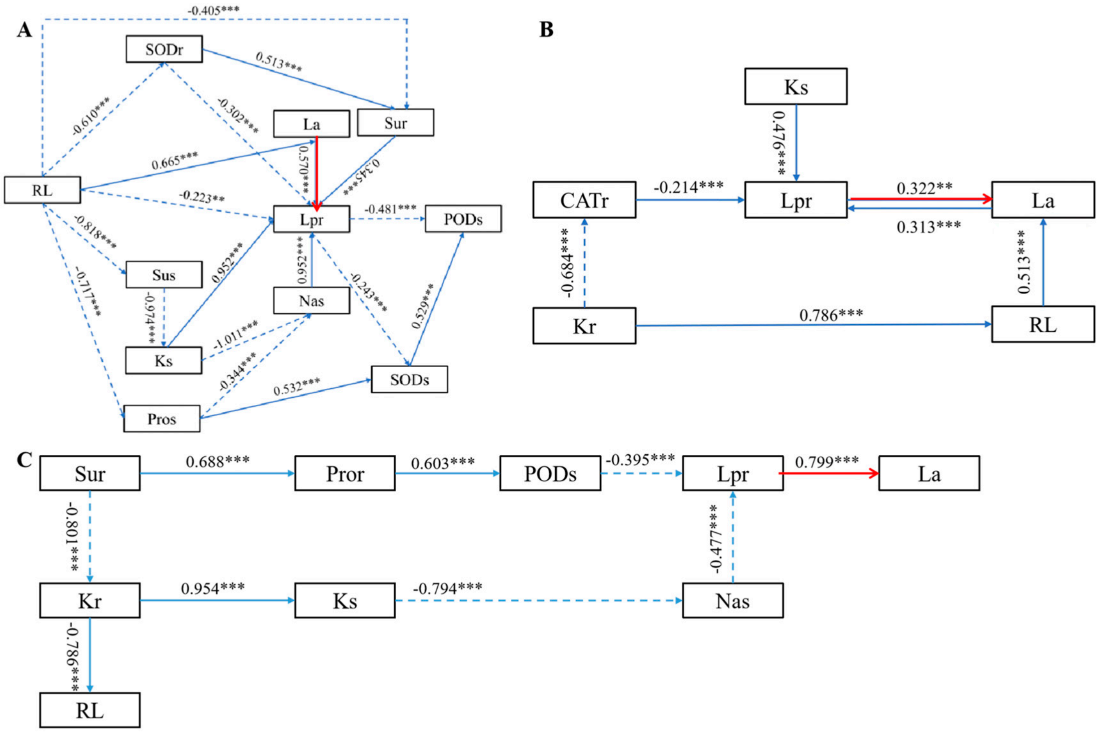

Figure S7. The path analysis of the relation of between root hydraulic conductivity and the physiological characteristics from no priming (A), water priming (B), and melatonin priming (C) under

PEG, NaCl, and PEG + NaCl treatment. Lpr = root hydraulic conductivity per plant; RL = root length; La = leaf area; SODr = SOD activity in root; SODs = SOD activity in shoot; PODs = POD activity in shoot; CATr = CAT activity in root; Kr = K^+ content in root; Ks = K^+ content in shoot; Nas = Na^+ content in shoot; Sus= soluble sugar content in shoot; Sur = soluble sugar content in root; Pros= proline content in shoot; Pror= proline content in root; ** indicates a significant difference at the level of P<0.01; *** indicates a significant difference at the level of P<0.001.

Table S1. Primers of QRT-PCR.

Primer	Primer sequence (5'-3')
QRT-*TaActin*-F	CACTGGAATGGTCAAGGCTG
QRT-*TaActin*-R	CTCCATGTCATCCCAGTTG
*QRT-TaNIP*1;08_*7D-F*	TGGCAGGAGGAGGCGATAAC
*QRT-TaNIP*1;08_*7D-R*	TCAGGAAGTAGGTCCCGAAAATC
*QRT-TaNIP*2;04a_*7D-F*	GGGGTTAGCAGTTGGTTCCG
*QRT-TaNIP*2;04a_*7D-R*	GCCAAGAAAGTAGATCCAGAGGC
*QRT-TaNIP*3;03_6*D-F*	CCGACGACGAGAATGAGAAATC
*QRT-TaNIP*3;03_6*D-R*	CCGACAGCACGATGAAGACG
QRT-TaNIP4;03_3D-F	GACGTTCCTGGTGGTGTTCTG
QRT-TaNIP4;03_3D-R	CCAGGGGAAGTAGCGGAA
QRT-TaPIP1;01_2*D-F*	CCATCATCTACAACAGGGAGCAC
QRT-TaPIP1;01_2*D-R*	CAGGAAGAACAGCAGCTTAGGAC
QRT-TaPIP2;40_7*D-F*	TTCGTCCTCGTCTACACCGTC
QRT-TaPIP2;40_7*D-R*	GCGTGCTCCCTGTTGTAGATG
*QRT-TaSIP*2;02_4*A-F*	GCCAAGGTCTCGCTCTCTGTC
*QRT-TaSIP*2;02_4*A-R*	CAATAACCGCCCCAATCAC
*QRT-TaTIP*4;03_5*A-F*	GCACATCACGGCGTTCAG
*QRT-TaTIP*4;03_5*A-R*	CACCACAAAGAGGAGGGAGAAG
QRT-Ta*SNAT*-F	ACTTGGTCGCCACACTACAT
QRT-TaSNAT-R	TCGACAAGGACGTCCCAAAT
QRT-TaCOMT-F	AAGGCAAGGTGGTAGTCGT
QRT-TaCOMT-R	CCTGCGTAGATGTAGGTGGTC
QRT-TaASMT1-F	TCTGCCAGGGCAATTGCTAA
QRT-TaASMT1-R	TTCAGGAAGAGAGCATCCGC
QRT-TaASMT2-F	CAACCTTATGGATAGACCTCGATGACAC
QRT-TaASMT2-R	GCTGATGGATGTGGCAATGATGGTA
QRT-*TaSIP1*-F	GGCAAGGACCGCTCCATCT
QRT-*TaSIP1*-R	GGTGTACTCGTGCATGACCC
QRT-*TaTPP7-5A*-F	GCCAAGTCCTCAAAGTCCAAG
QRT-*TaTPP7-5A*-R	GTGCTTCGTCTCCTCTATCAG
QRT-*TaTPP7-5B*-F	CCCCTGCTCATCCGAACAA
QRT-*TaTPP7-5B*-R	AAGGGCGGTTAATTAGAACACA
QRT-*TaTPP7-5D*-F	AAGGTGTTTGATTTCGTGAAGC
QRT-*TaTPP7-5D*-R	CTTCTATCATGGGCAGGAACTC
QRT-*TaCIPK10*-F	TCTGGTGGAAATCAGGAAGACC
QRT-*TaCIPK10*-R	TTGCCAAGCCCAAACAATGTC
QRT-*TaCIPK27*-F	CCGGCGCTCAAGGACATCG
QRT-*TaCIPK27*-R	ATTACGGCCGGATAGCAACAGT
